# Supplementary material for: Atomic-precision Pt6 nanoclusters for enhanced hydrogen electro-oxidation
Source: Nat Commun. 2022 Mar 24;13:1596. doi: 10.1038/s41467-022-29276-7 (PMC8948276; doi:10.1038/s41467-022-29276-7)
Supplement: Supplementary file 1 — Supplementary Information [file 41467_2022_29276_MOESM1_ESM.pdf]

Supplementary Information for

Atomic-precision Pt_6 nanoclusters for enhanced hydrogen electro-oxidation

Wang et al.

Supplementary Figures

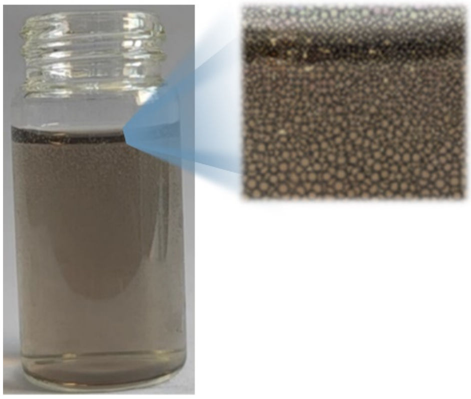

Supplementary Figure 1. A typical image of the suspension solution containing Pt ions and $NaBH_4$. The image shows the fast release of abundant H_2 bubbles (shown in the upper solution).

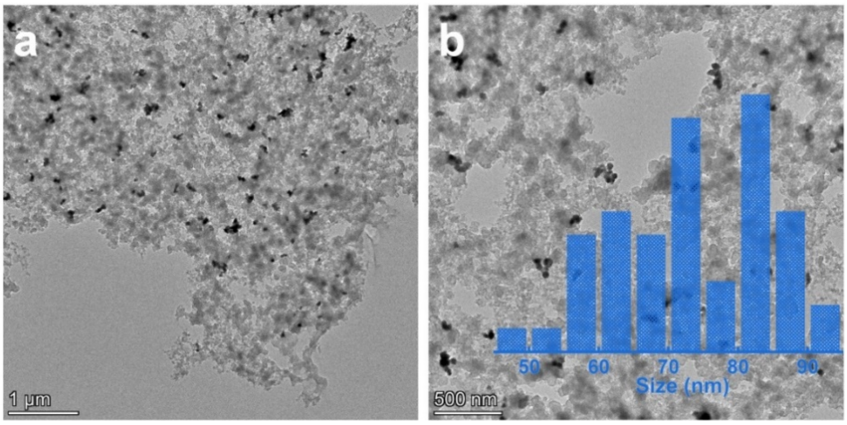

Supplementary Figure 2. Morphological observation of PtNPs/C-$NaBH_4$. (a) Low- and (b) high-magnification TEM images of PtNPs/C-$NaBH_4$. The inset of b shows the size distribution of the Pt particles.

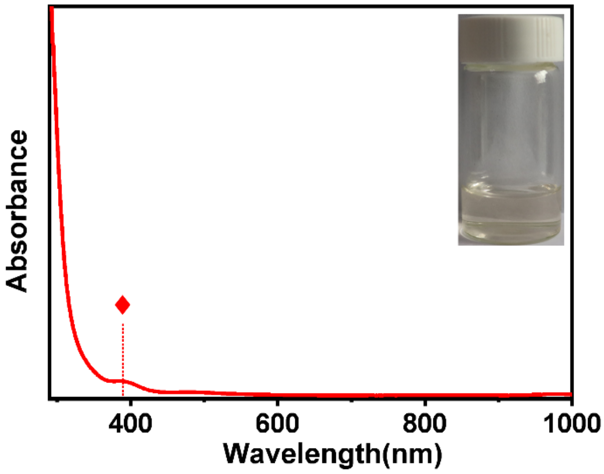

Supplementary Figure 3. UV-vis absorption spectrum and the photographic image of the Pt_1 species. It presents a distinctive peak at 390 nm in the UV-vis spectrum. The inset shows the photographic image of of the Pt_1 species suspension.

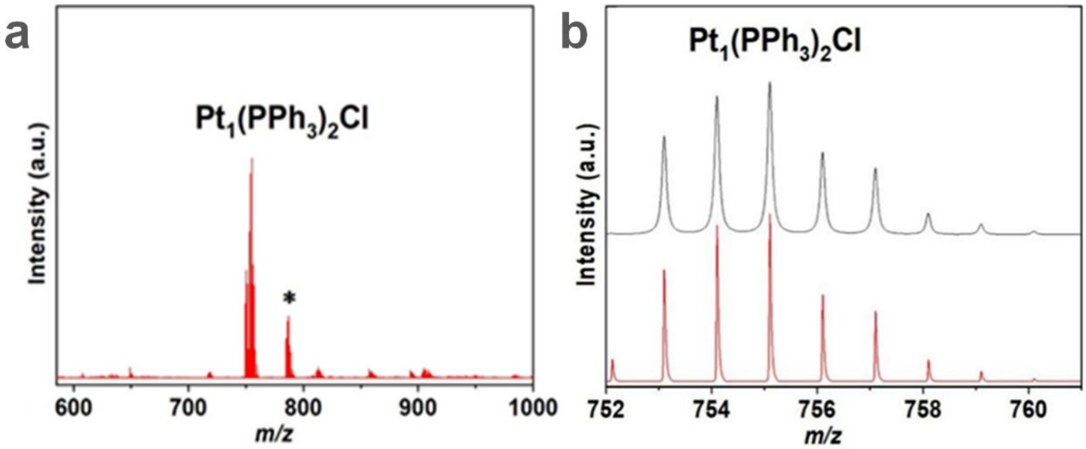

Supplementary Figure 4. Experimentally obtained ESI-MS spectrum and simulated result of the Pt_1 species. (a) ESI-MS spectrum of the Pt_1 species, i.e., $Pt_1(PPh_3)_2Cl$ (measured in positive mode). The species labeled by * is $(PPh_3)_3$. (b) Experimentally obtained (red curve) and simulated (black curve) isotope patterns of the $Pt_1(PPh_3)_2Cl$.

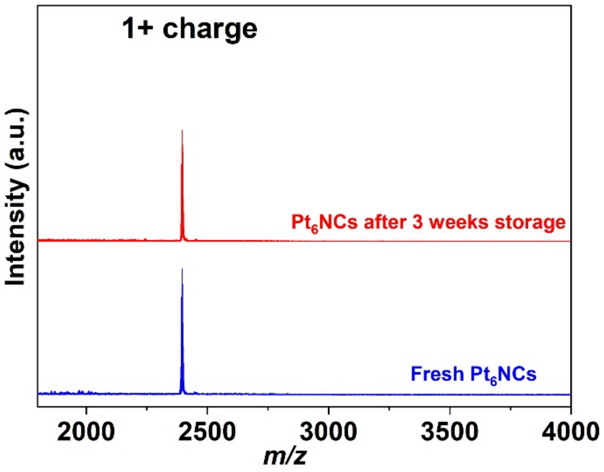

Supplementary Figure 5. Structural stability analysis. ESI-MS spectra of the Pt_6NCs freshly prepared (blue curve) and after three weeks storage (red curve) at 4 ℃ without N_2 protection do not change significantly, indicating good structural stability.

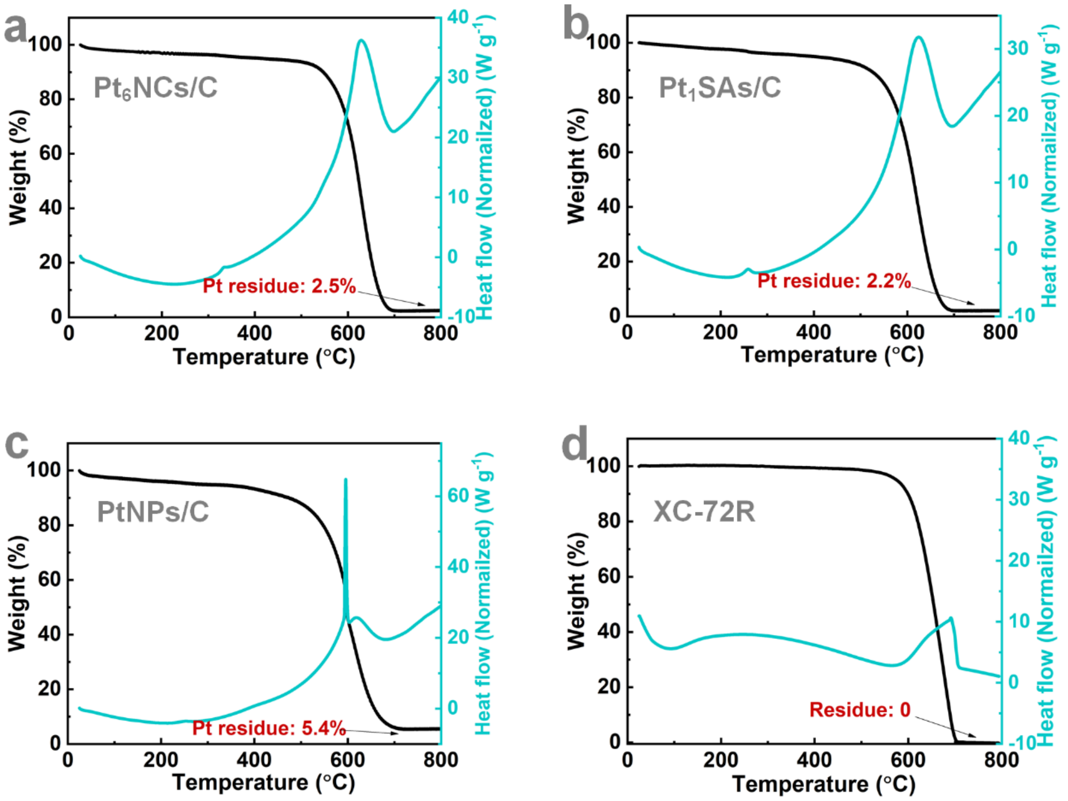

Supplementary Figure 6. Pt loading of different samples obtained by TGA. TGA of (a) Pt_6NCs/C, (b) Pt_1SAs/C, (c) PtNPs/C, and (d) XC-72R under air atmosphere.

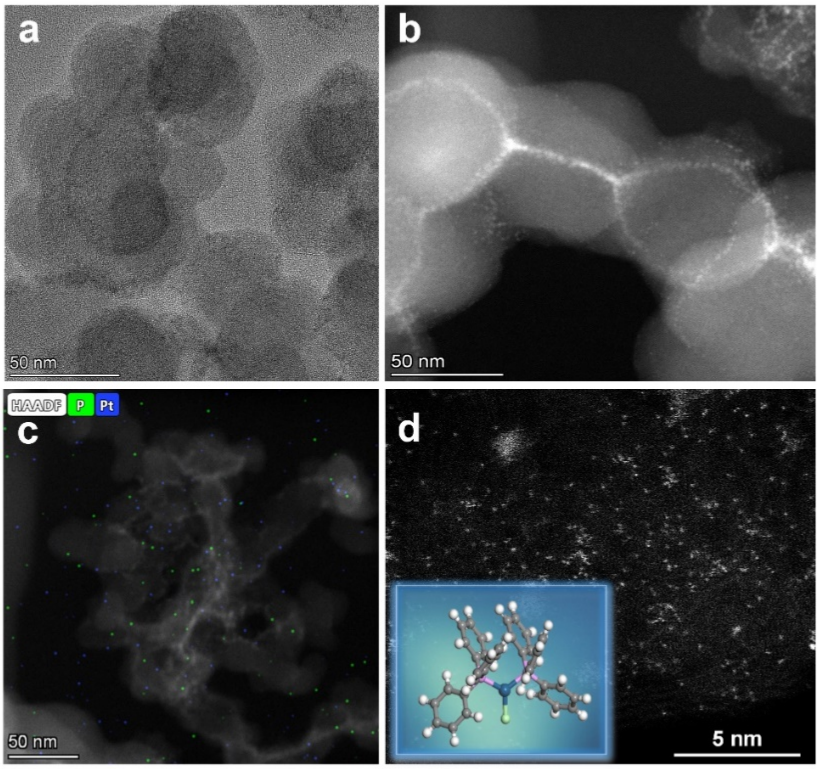

Supplementary Figure 7. Morphological observation of Pt_1SAs/C. (a) TEM image. (b) HAADF-STEM image. (c) Corresponding element map showing distributions of Pt and P. (d) Aberration-corrected HAADF-STEM image; inset: the ideal structure model of a Pt_1SAs. The gray, white, blue, pink, and reseda spheres represent C, H, Pt, P, and Cl atoms, respectively.

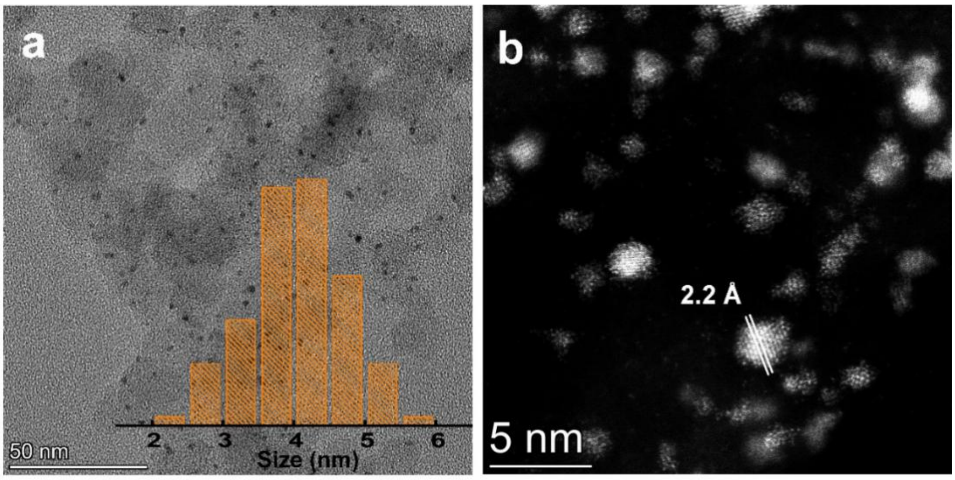

Supplementary Figure 8. Morphological observation of PtNPs/C. (a) TEM image; inset: size distribution of the Pt NPs. (b) HAADF-STEM image.

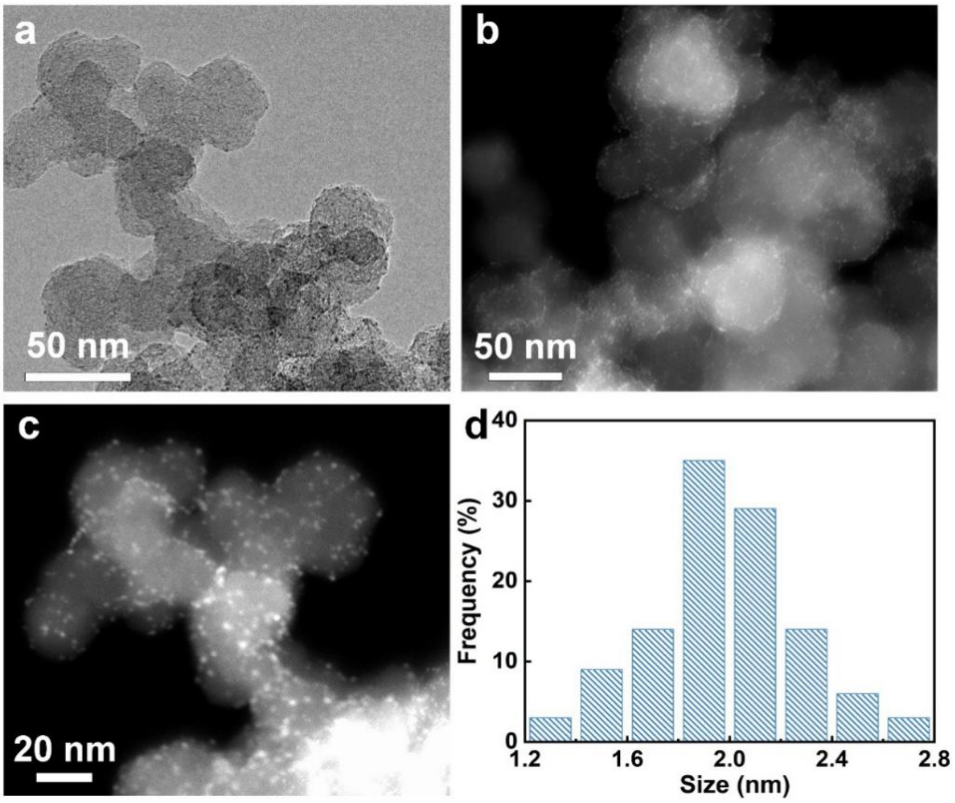

Supplementary Figure 9. Morphological observation of GSH-PtNCs/C. (a) TEM image. (b and c) HAADF-STEM images. (d) The size distribution histograms of Pt clusters.

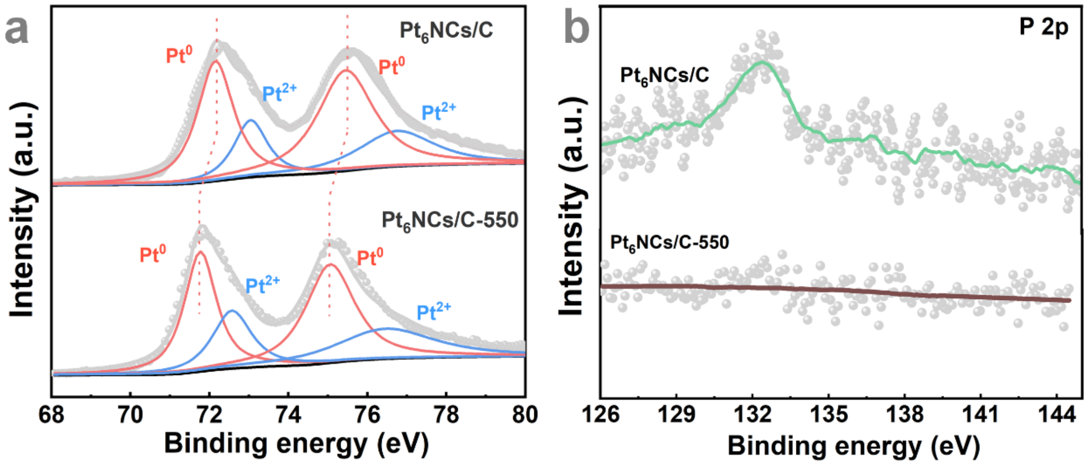

Supplementary Figure 10. XPS characterization. (a) High-resolution XPS Pt 4*f* spectra of Pt_6NCs/C and Pt_6NCs/C-550. (b) High-resolution XPS P 2*p* spectra comparison of Pt_6NCs/C and Pt_6NCs/C-550.

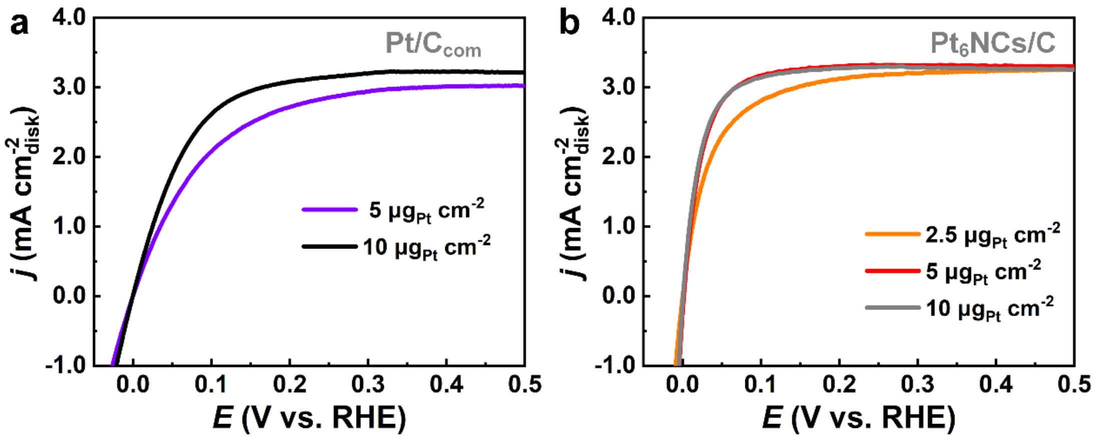

Supplementary Figure 11. The effect of catalyst loadings on HOR polarization curves. HOR polarization curves of (a) Pt/C_{com} and (b) Pt_6NCs/C with different loading densities at a scan rate of 5 mV s^{-1} and a rotating speed of 2500 rpm in H_2-saturated 0.1 M KOH.

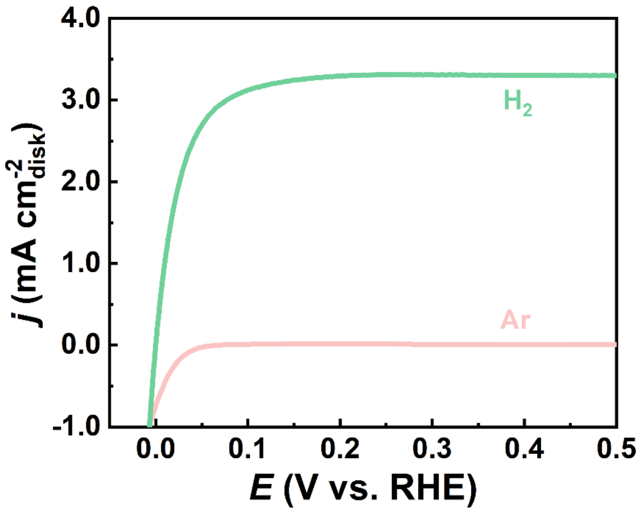

Supplementary Figure 12. HOR polarization curves of Pt_6NCs/C in H_2 and Ar-saturated 0.1 M KOH. By comparing the polarization curves of Pt_6NCs/C in different gas-saturated electrolytes, the speedy current response under H_2 and the sluggish current response under Ar can confirm that the anode current derives from the oxidation of hydrogen rather than that of other species. However, we also observed the current peak in an Ar-saturated solution at a voltage below 0 V, which can be ascribed to the hydrogen evolution reaction of the Pt_6NCs/C.

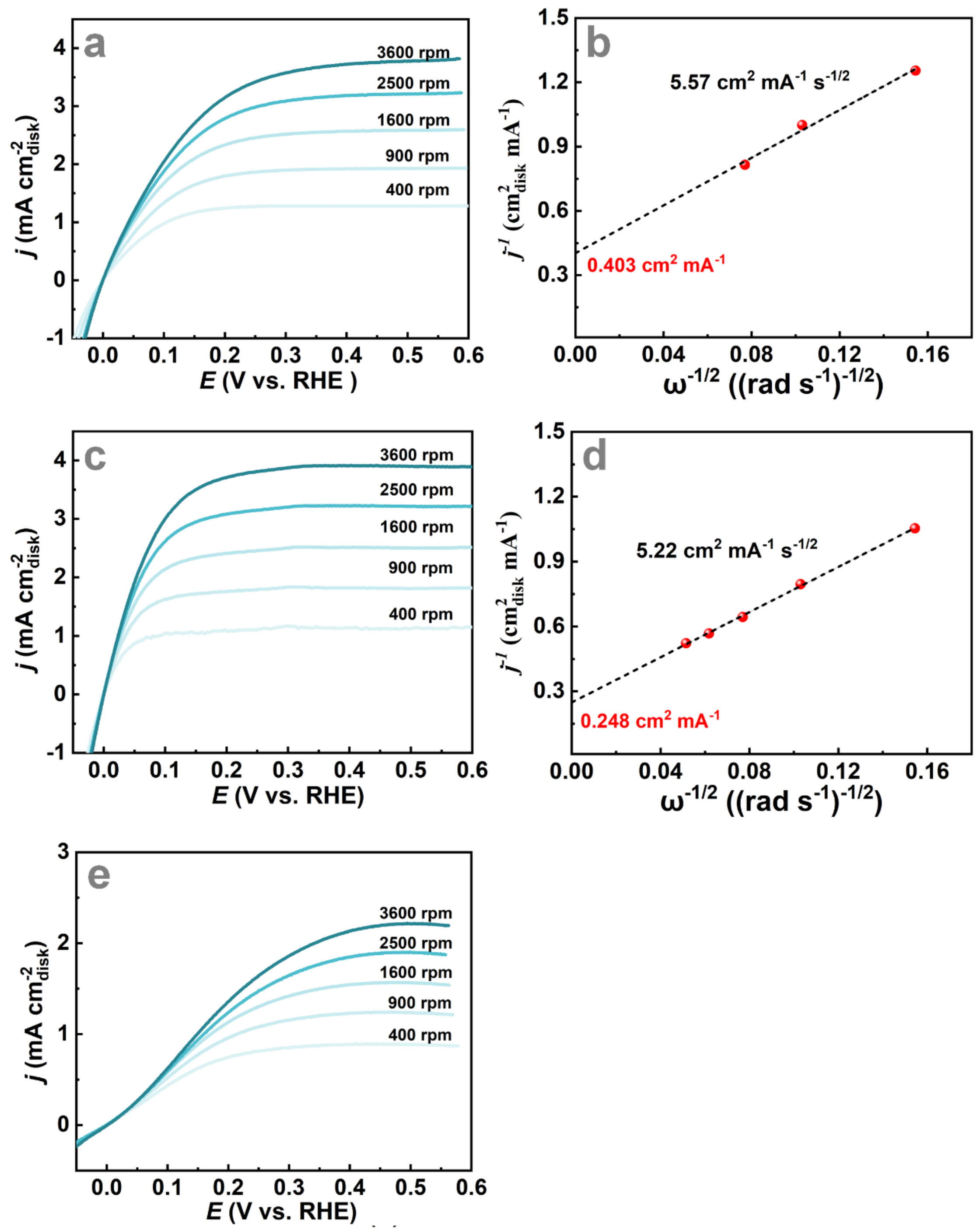

Supplementary Figure 13. HOR activities of different catalysts. HOR polarization curves of (a) PtNPs/C, (c) Pt/C_{com}, and (e) Pt_1SAs/C catalyst at various rotating speeds. The Koutecky-Levich plot of (b) PtNPs/C and (d) Pt/C_{com} catalyst at an overpotential of 50 mV vs. RHE.

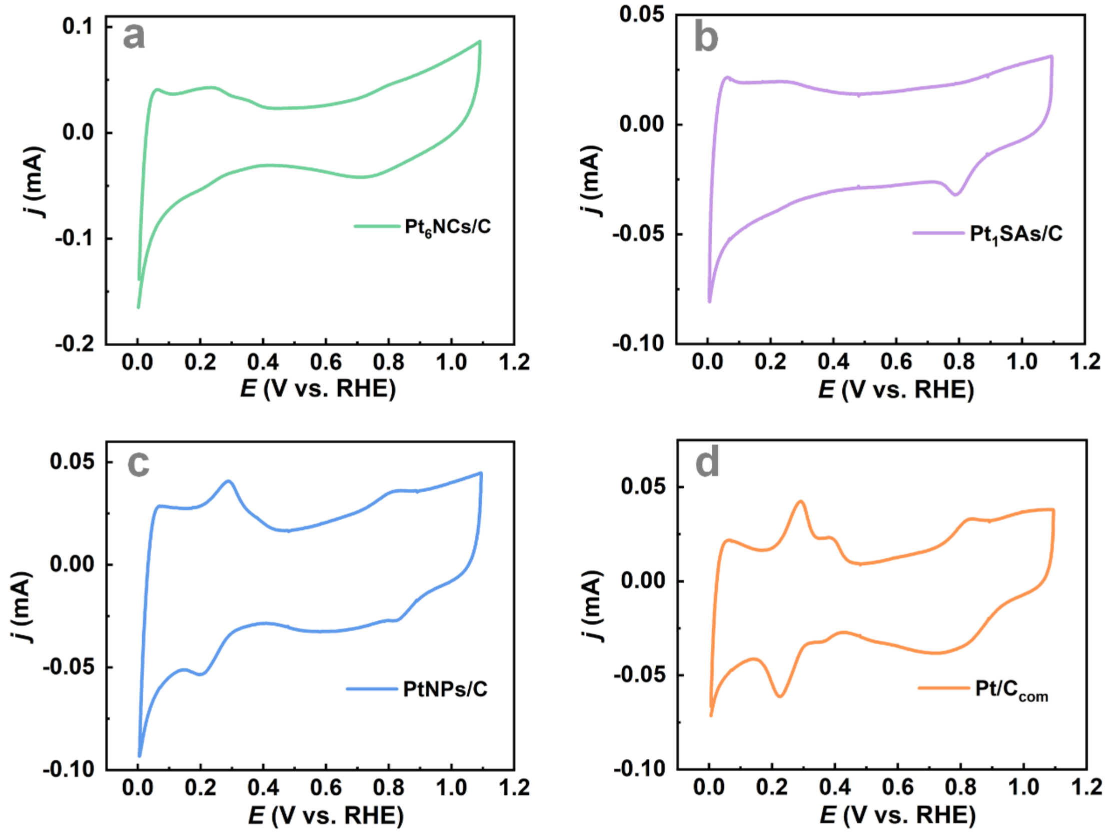

Supplementary Figure 14. ECSA measurements. CV curves of (a) Pt_6NCs/C, (b) Pt_1SAs/C, (c) PtNPs/C, and (d) Pt/C_{com} catalysts conducted in Ar-saturated 0.1 M KOH at a scan rate of 50 mV s^{-1}. The hydrogen underpotential deposition (H_{upd}) desorption peaks of Pt/C_{com} located at 0.293 V and 0.386 V are assigned to the two lattice planes of Pt (1 1 0) and Pt (1 0 0), respectively. In contrast, the H_{upd} desorption peaks of Pt_6NCs/C and Pt_1SAs/C are not obvious, inferring the absence of a corresponding lattice structure.

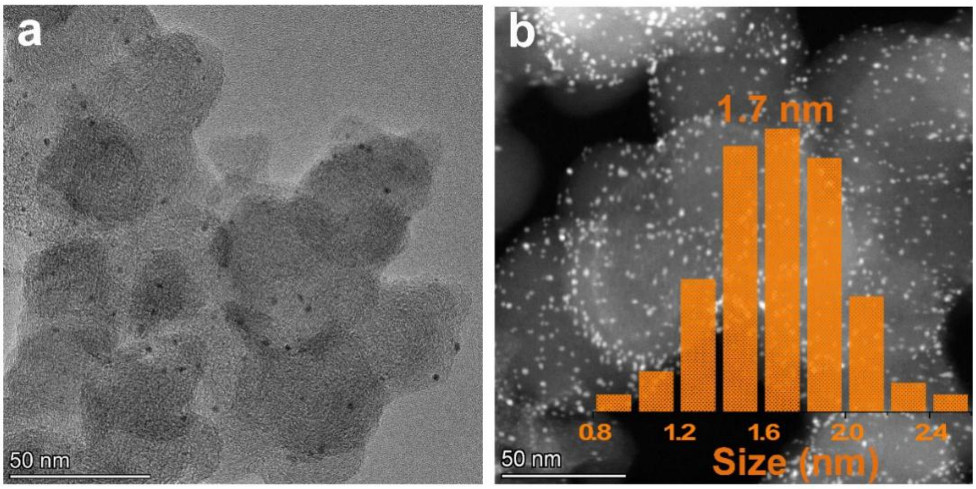

Supplementary Figure 15. Morphological observation of Pt_6NCs/C-550. (a) TEM image. (b) HAADF-STEM image; inset: size distribution of the Pt clusters.

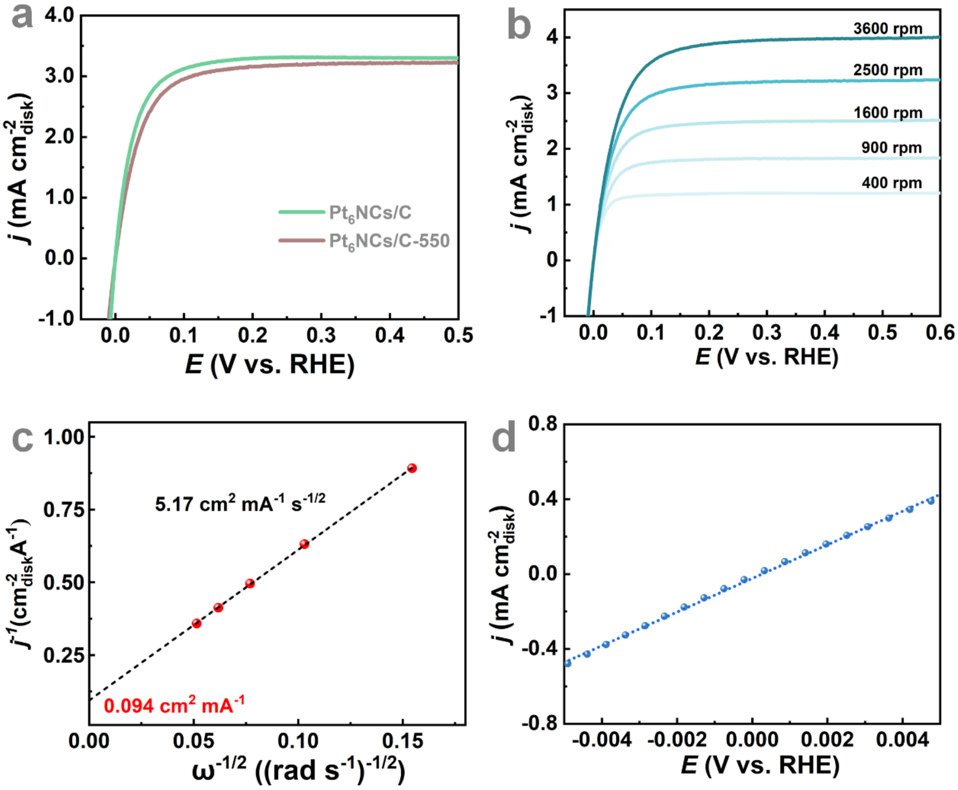

Supplementary Figure 16. HOR activity of Pt_6NCs/C-550. (a) HOR polarization curves of Pt_6NCs/C-550 and Pt_6NCs/C catalysts in H_2-saturated 0.1 M KOH solutions with the rotation speed of 2500 rpm at a scan rate of 5 mV s^{-1}. (b) HOR polarization curves of Pt_6NCs/C-550 at different rotation rates. (c) the Koutecky-Levich plot of Pt_6NCs/C-550 catalyst at an overpotential of 50 mV (vs. RHE). (d) Linear current potential region around the equilibrium potential of Pt_6NCs/C-550. The dotted lines indicate the linear fitting of the data.

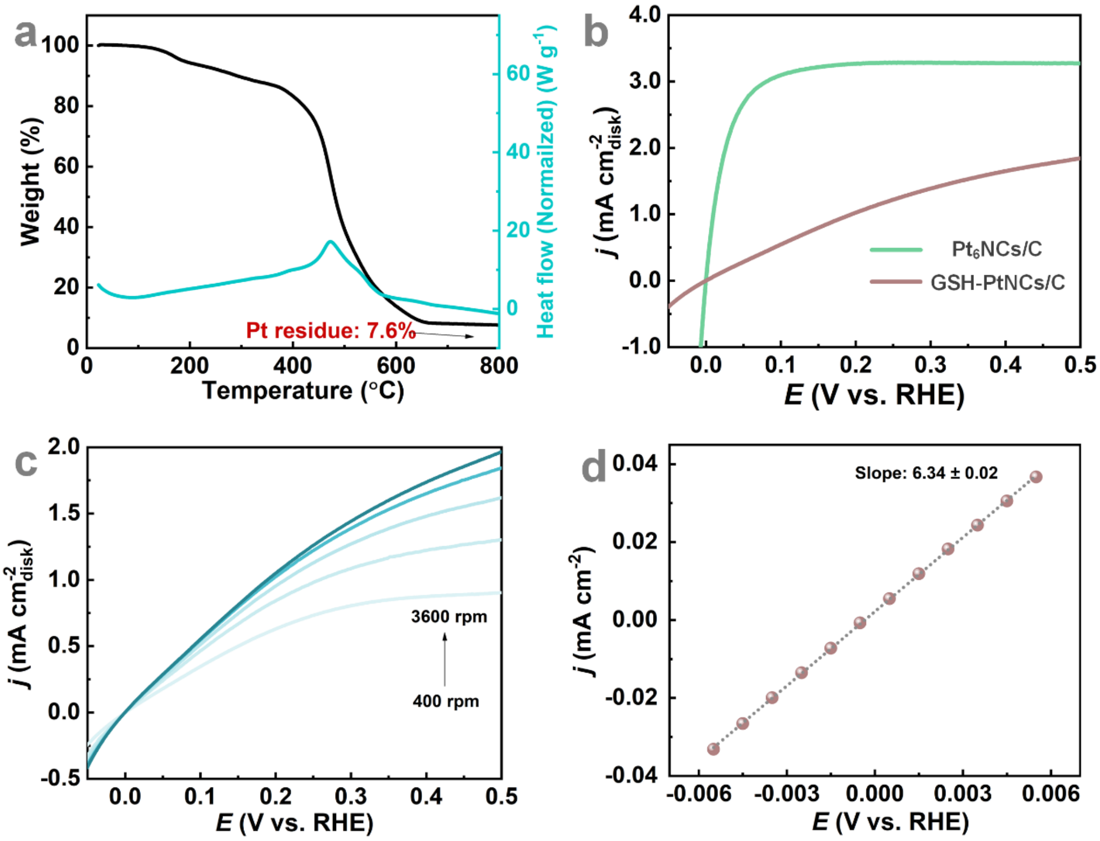

Supplementary Figure 17. Pt loading analysis and HOR activity of GSH-PtNCs/C. (a) TGA under air atmosphere. The Pt loading of GSH-PtNCs/C was measured to be 7.6 wt.% by TGA under the air. (b) HOR polarization curves of Pt_6NCs/C and GSH-PtNCs/C catalysts in H_2-saturated 0.1 M KOH solutions with the rotation speed of 2500 rpm at a scan rate of 5 mV s^{-1}. (c) HOR polarization curves of GSH-PtNCs/C at different rotation rates. (d) The linear current potential region around the equilibrium potential of HOR/HER of GSH-PtNCs/C. The j_0 is also determined from the linear fitting of the micro-polarization region that deviates only several millivolts from the equilibrium potential (from -5 to 5 mV vs. RHE). The $j_{0,m}$ of GSH-PtNCs/C was calculated to be only 32.5 A g^{-1}.

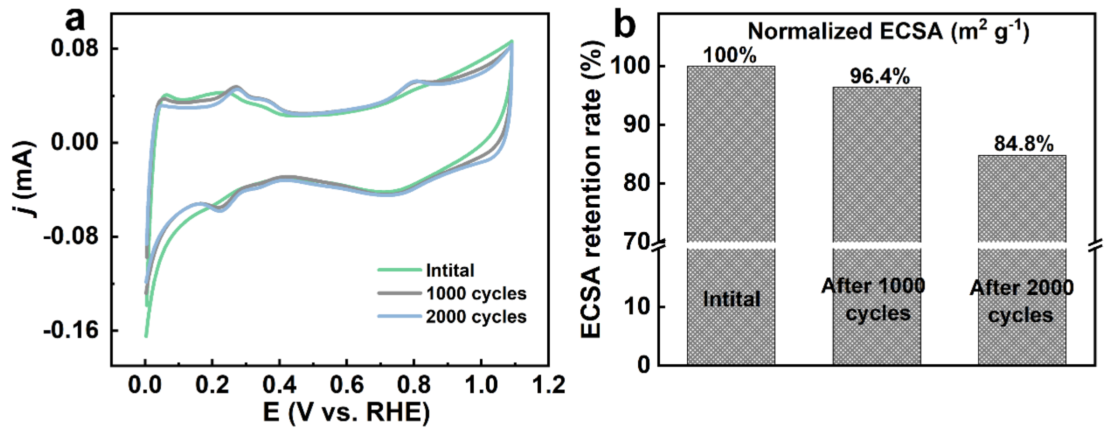

Supplementary Figure 18. Stability test results of Pt_6NCs/C. (a) CV plots. (b) Change of ECSA.

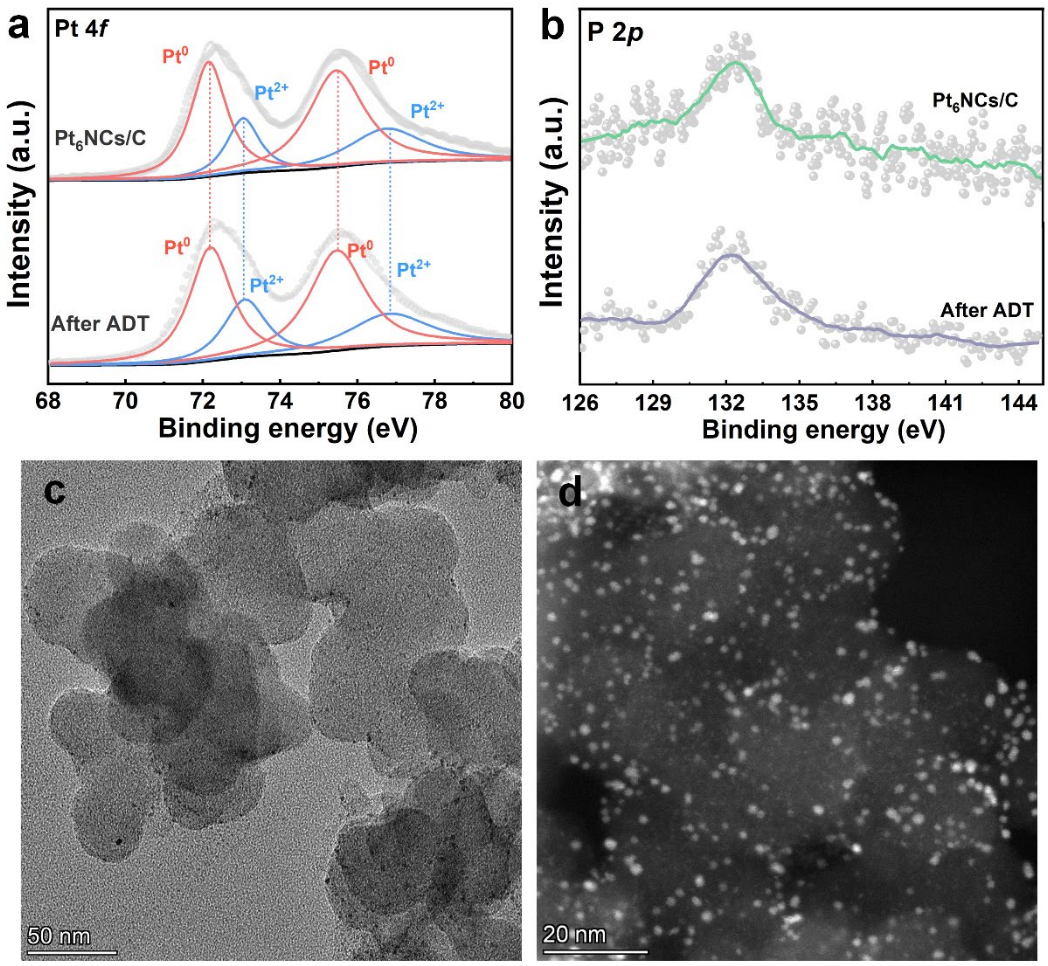

Supplementary Figure 19. XPS and morphological characterization of Pt_6NCs/C after ADT. (a) High-resolution XPS Pt 4*f* spectra before and after ADT. (b) High-resolution XPS P 2*p* spectra before and after ADT. (c) TEM and (d) HAADF-STEM images after ADT.

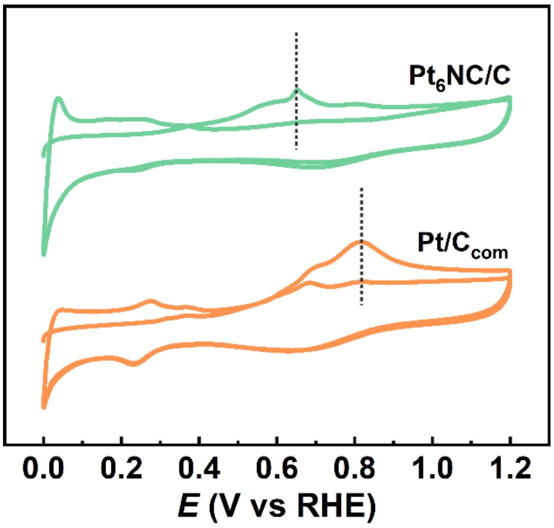

Supplementary Figure 20. CO-stripping curves. CO-stripping measurements on Pt_6NC/C and Pt/C_{com} catalyst in 0.1 M KOH electrolyte.

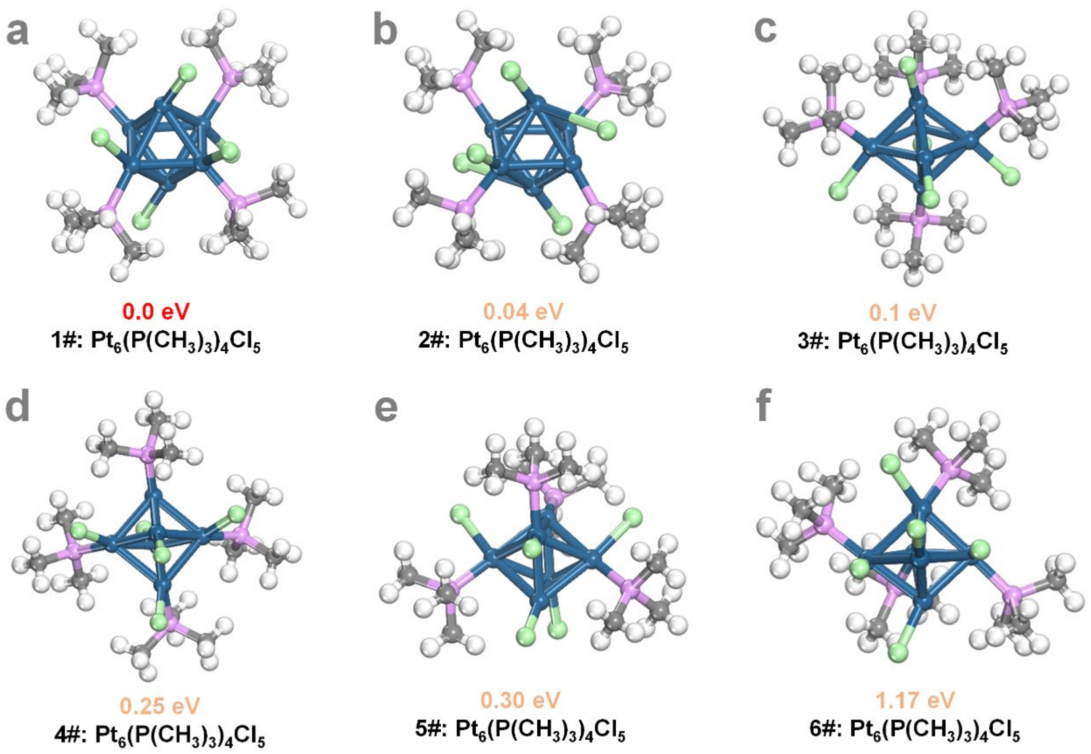

Supplementary Figure 21. Computed structures of the Pt_6NCs with different coordination configurations. (a) 1# coordination configuration. (b) 2# coordination configuration. (c) 3# coordination configuration. (d) 4# coordination configuration. (e) 5# coordination configuration. (f) 6# coordination configuration. Bottom values indicate the energy differences relative to the most stable structure. The gray, white, blue, pink, and reseda spheres represent C, H, Pt, P, and Cl atoms, respectively.

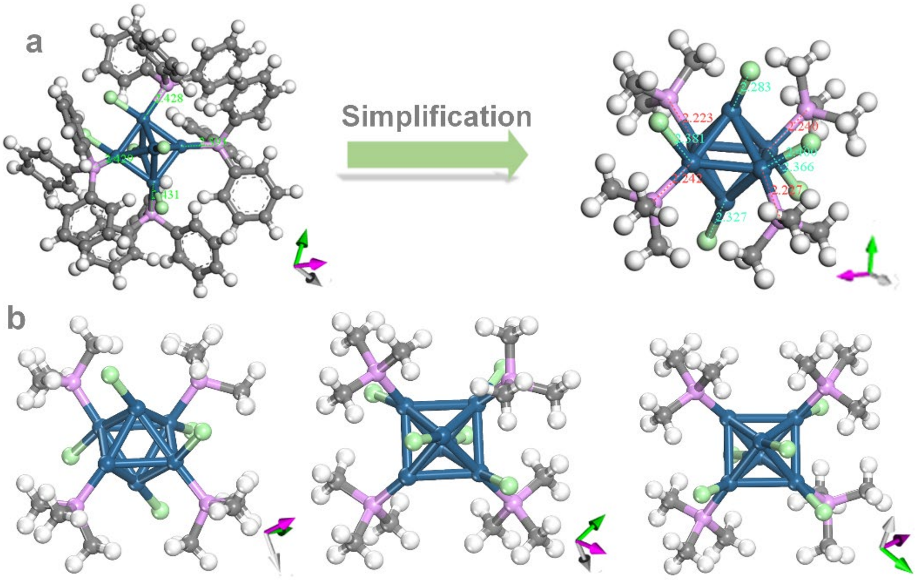

Supplementary Figure 22. Constructed model of Pt_6NCs. (a) Constructed model before and after simplification. (b) Simplified models from different perspectives. The gray, white, blue, pink, and reseda spheres represent C, H, Pt, P, and Cl atoms, respectively.

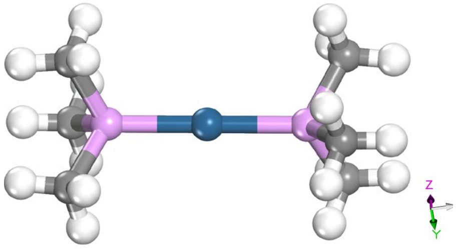

Supplementary Figure 23. Constructed models of Pt_1SAs. The gray, white, blue, and pink spheres represent C, H, Pt, and P atoms, respectively.

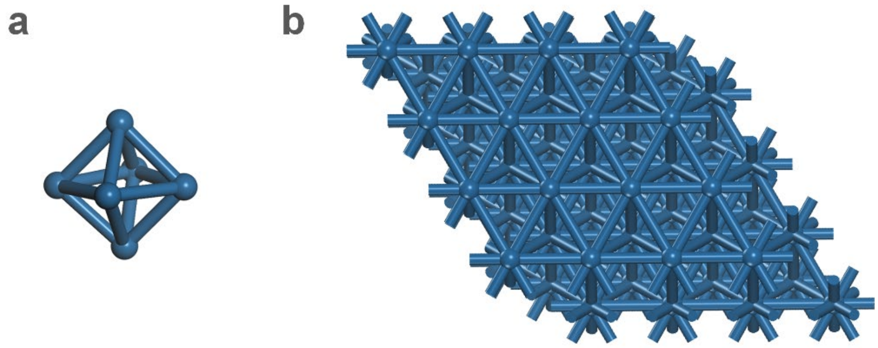

Supplementary Figure 24. Constructed models. (a) Pt_6NCs-550/C. (b) PtNPs. Pt(1 1 1) here is a simplified structure of PtNPs. The blue spheres represent Pt.

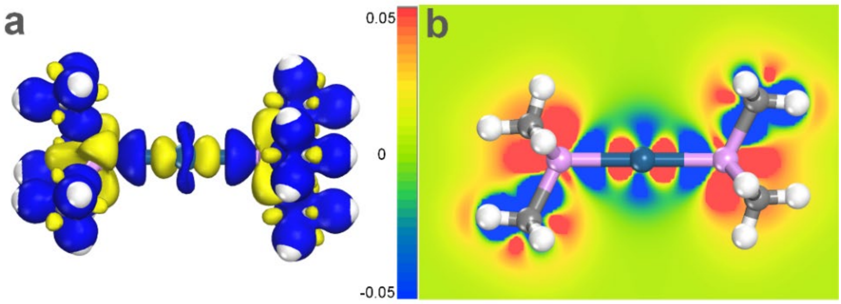

Supplementary Figure 25. The 3D contour plot and slice perspective of charge density distribution for Pt_1SAs. (a) The 3D contour plot of charge distribution. Light yellow and blue areas denote charge density depletion and accumulation, respectively. (b) The slice perspective of differential charge density distribution. In the electron density difference maps, the red and blue colors refer to the positive (0.05 e Å^{-3}) and negative (-0.05 e Å^{-3}) values, respectively. The gray, white, blue, and pink spheres represent C, H, Pt, and P atoms, respectively.

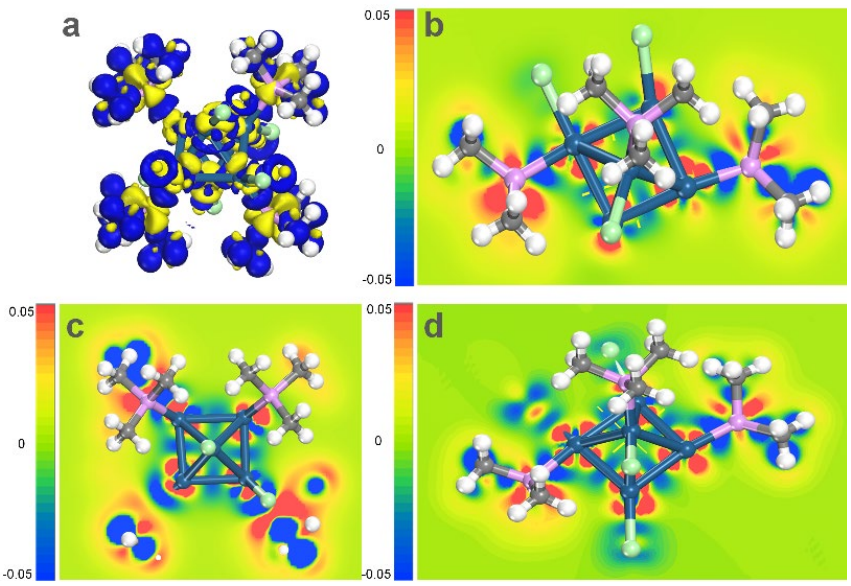

Supplementary Figure 26. The 3D contour plot and slice perspective of charge density distribution for Pt_6NCs. (a) The 3D contour plot of charge distribution. Light yellow and blue areas denote charge density depletion and accumulation, respectively. (b-d) The slice perspective of differential charge density distribution from different perspectives. In the electron density difference maps, the red and blue colors refer to the positive (0.05 e Å^{-3}) and negative (-0.05 e Å^{-3}) values, respectively. The gray, white, blue, pink, and reseda spheres represent C, H, Pt, P, and Cl atoms, respectively.

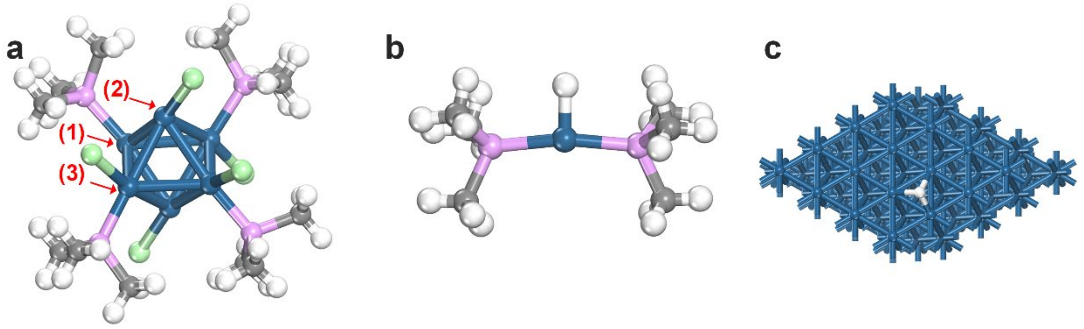

Supplementary Figure 27. Models of H adsorption on catalysts. (a) Pt_6NCs. (b) Pt_1SAs. (c) PtNPs. Numbers in panel (a) represent different H adsorption sites. The gray, white, blue, pink, and reseda spheres represent C, H, Pt, P, and Cl atoms, respectively.

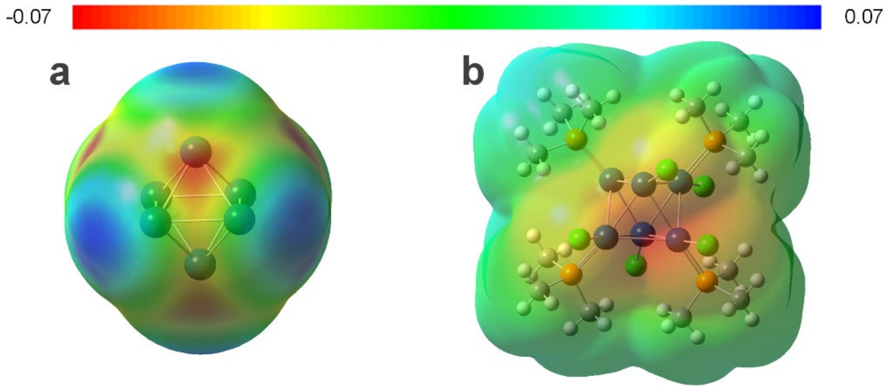

Supplementary Figure 28. Surface electrostatic potential analysis. Surface electrostatic potentials of (a) bare Pt_6NC and (b) ligand-protected Pt_6NC. Surface electrostatic potential mapped on the isosurface (0.0001 a.u.) of electronic density.

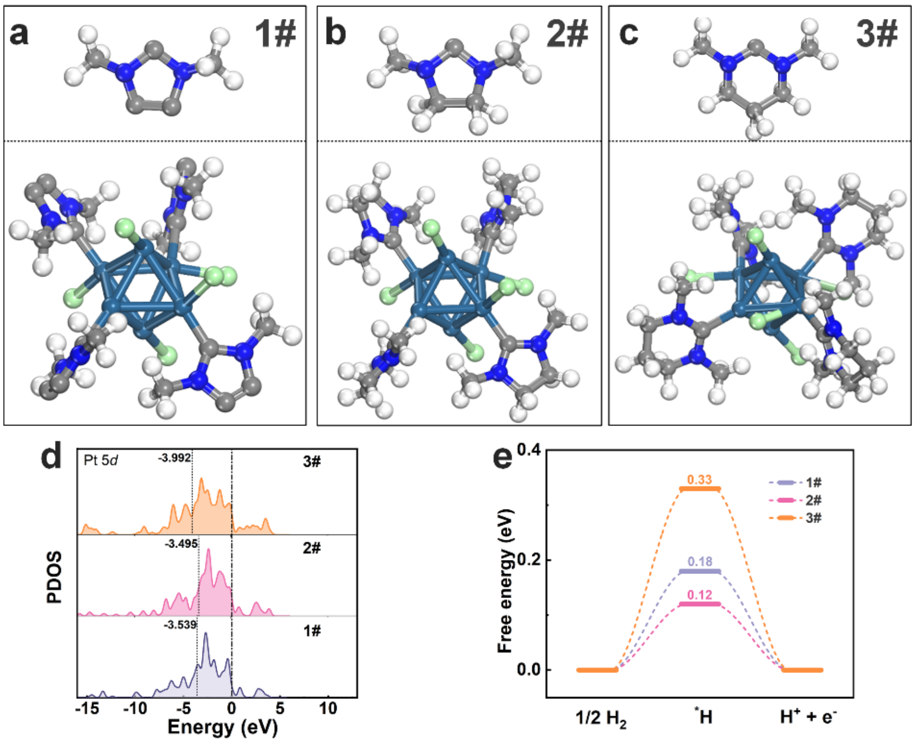

Supplementary Figure 29. Structure-activity analysis of N-heterocyclic carbene (NHC) ligand-protected Pt_6 NCs. (a-c) Structures of Pt_6 NCs protected by different NHC ligands and their NHC ligands. (d) The PDOSs of Pt 5*d* in 1#, 2#, and 3# models (each *d*-band center is marked by a dashed line) with the Fermi level aligned at 0 eV. (e) ΔG_{*H} on 1#, 2#, and 3# Pt_6 NCs.

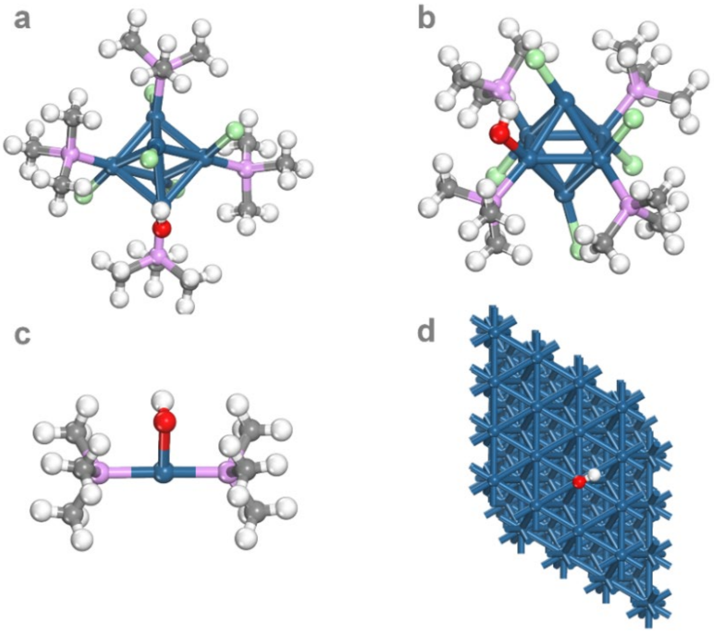

Supplementary Figure 30. Models of OH adsorption on catalysts. (a, b) Pt_6NCs. (c) Pt_1SAs. (d) PtNPs. The gray, red, white, blue, pink, and reseda spheres represent C, O, H, Pt, P, and Cl atoms, respectively.

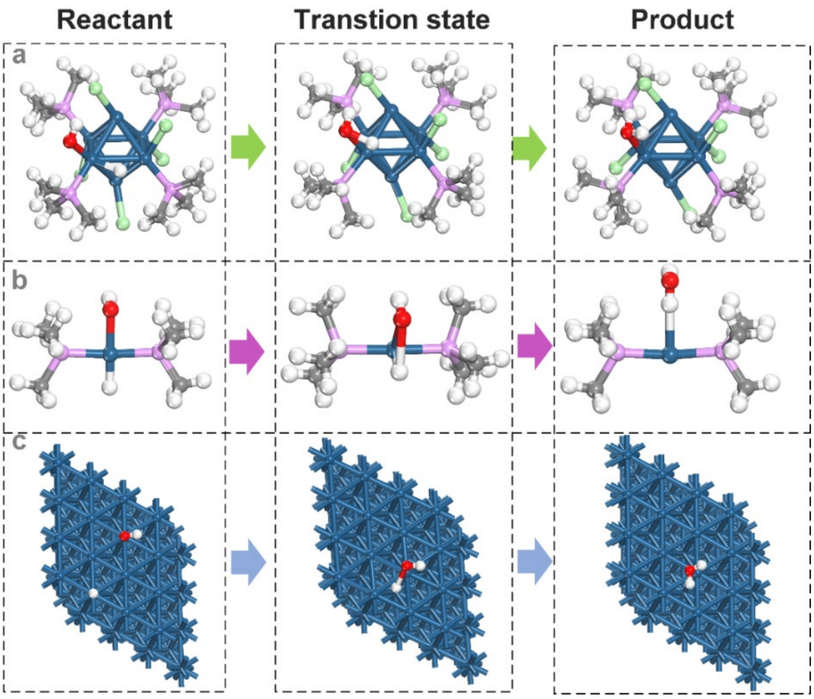

Supplementary Figure 31. Models of H_2O formation process on catalysts. (a) Pt_6NCs. (b) Pt_1SAs. (c) PtNPs. The gray, red, white, blue, pink, and reseda spheres represent C, O, H, Pt, P, and Cl atoms, respectively.

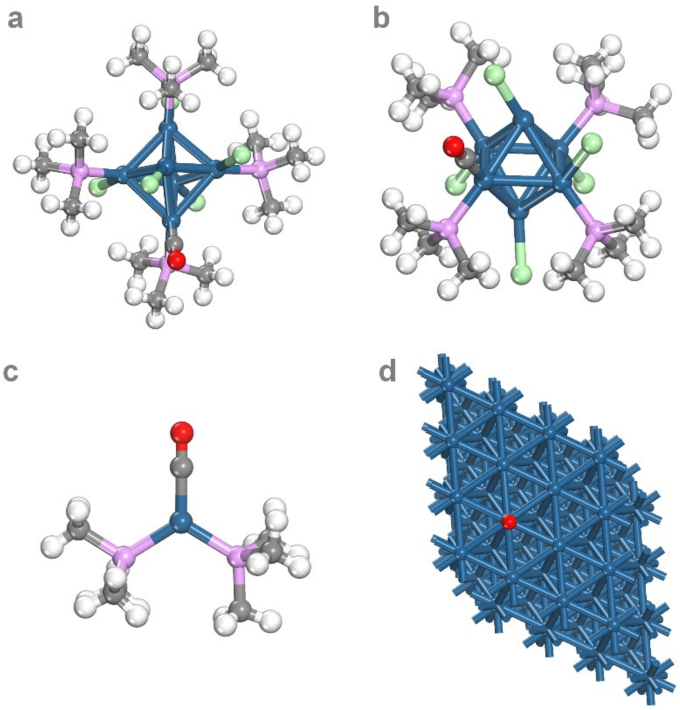

Supplementary Figure 32. Models of CO adsorption on catalysts. (a, b) Pt_6NCs. (c) Pt_1SAs. (d) PtNPs. The gray, red, white, blue, pink, and reseda spheres represent C, O, H, Pt, P, and Cl atoms, respectively.

Supplementary Tables

Supplementary Table 1. Benchmark HOR activities and the relevant parameters of different catalysts.

Catalyst	Catalyst loading [$\mu g_{cat.}$ cm^{-2}_{disk}]	$j_{k,m}$ @η =50 mV [A $g^{-1}_{cat.}$]	ECSA [m^2 $g^{-1}_{cat.}$]	$j_{0,s}$ [mA $cm^{-2}_{cat.}$]	Reference
Pt_6NCs/C	5	3658	41.8	1.546	This work
Pt_1SAs/C	5	/	14.8	0.149	
PtNPs/C	5	496	36.9	0.446	
Pt/C_{com}	10	402	32.2	0.351	
$Pd_3Fe@Pt/C$	1.87	661	46.9	0.511	1
$Pd_3Co@Pt/C$	1.87	685	43.9	0.570	
$PtNb/NbO_x$-C	/	360	45	0.8	2
Rh_2Sb NBs/C	6.4	3254	1.14 (cm^2)	0.506	3
$Pt_{0.25}Ru_{0.75}$/N-C	50	1654	117.3	1.41	4
Acid-PtNi/C	10	474	25.1	1.89	5
Pt/Cu NWs	86.02	650	35.9	2.1	6
$Pt_{0.8}Ru_{0.2}/C$	7.09	696	49.0	1.42	7
$Ru_{0.96}Pt_{0.04}$	7.01	2580	10.5	/	8
Pt_7Ru_3 NW	10	/	0.176 (cm^2)	0.493	9
PtRh	25.5	322	32.1	1.25	10
$PtRu/Mo_2C$	13	239	/	0.17	11
$PtRu/Mo_2C$-TaC	13	403	/	0.28	
$PtRu/Mo_2C$-W_2C	13	236	/	0.19	

Supplementary Discussion

Supplementary Discussion 1: the origin of the ligand effect.

Owing to the difficulties in distinguishing the effects of Cl ligands and PPh_3 ligands alone on HOR activity through an experimental approach, we resorted to DFT simulations to distinguish the contributions of Cl ligands and PPh_3 ligands to HOR activity. In the Pt core of Pt_6NCs, there are three types of Pt atoms with different coordination environments, namely (1) Pt-P, (2) Pt-Cl, and (3) P-Pt-Cl (Supplementary Fig. 27a). It is found that the ΔG_{*H} of site 1 (-0.07 eV) for Pt_6NCs is very close to the ideal value (ΔG_{*H} = 0) for HOR. In comparison, the ΔG_{*H} values of site 2 (+0.24 eV) and site 3 (+0.46 eV) reveal larger deviation from the ideal value. These results indicate that the PPh_3 ligands can significantly optimize the HBE of Pt compared to the Cl ligands.

Supplementary Discussion 2: surface electrostatic potential analysis.

Supplementary Fig. 28 shows the surface electrostatic potential mapped onto the total electron density of the bare Pt_6NCs and ligand-protected Pt_6NCs. The red regions of the molecular surface exhibit a low electrostatic potential, indicating nucleophilic properties, while the high electrostatic potential (blue color) of the molecular surface suggests the electrophilic properties. The electrostatic potential surface for bare Pt_6NCs confirms the higher electron affinities of the Pt atoms at the tetrahedral vertices (Supplementary Fig. 28a). The positive electrostatic potential represented by the dark blue regions delimits electrophilic zones, where the cluster is susceptible to chemical bonding by nucleophiles such as PPh_3 and Cl. As discussed above, apical Pt sites are the most stable sites for PPh_3 and Cl binding which provide strong Pt interaction and stabilize the geometry. When the geometry of the Pt_6 crystal is maintained, the charges coming from the unshared pair of electrons of PPh_3 distribute over the Pt core, and a slight charge redistribution occurs at Pt-P bonding, creating nucleophilic areas at the center sites of the cluster, as shown by the red regions of the electrostatic potential of Pt_6NCs in Supplementary Fig. 28b. In this red negative potential region, electrophiles

or positively charged species (such as hydrogen intermediates) can approach the cluster core. On the other hand, Pt_6NCs present a limited negative electrostatic potential sterically obstructed by ligands bonded to Pt atoms, resulting in a reduced desorption energy barrier for *H in the Volmer step.

Supplementary References

1. Zhao T, *et al.* Electronic structure and oxophilicity optimization of mono-layer Pt for efficient electrocatalysis. *Nano Energy* **74**, 104877 (2020).
2. Ghoshal S, *et al.* Tuning Nb–Pt interactions to facilitate fuel cell electrocatalysis. *ACS Catal.* **7**, 4936-4946 (2017).
3. Zhang Y, *et al.* Atomically isolated Rh sites within highly branched Rh_2Sb nanostructures enhance bifunctional hydrogen electrocatalysis. *Adv. Mater.* **33**, 2105049 (2021).
4. Cong Y, Chai C, Zhao X, Yi B, Song Y. $Pt_{0.25}Ru_{0.75}$/N-C as highly active and durable electrocatalysts toward alkaline hydrogen oxidation reaction. *Adv. Mater. Interfaces* **7**, 2000310 (2020).
5. Lu S, Zhuang Z. Investigating the influences of the adsorbed species on catalytic activity for hydrogen oxidation reaction in alkaline electrolyte. *J. Am. Chem. Soc.* **139**, 5156-5163 (2017).
6. Alia SM, Pivovar BS, Yan Y. Platinum-coated copper nanowires with high activity for hydrogen oxidation reaction in base. *J. Am. Chem. Soc.* **135**, 13473-13478 (2013).
7. St. John S, Atkinson RW, Unocic RR, Zawodzinski TA, Papandrew AB. Ruthenium-alloy electrocatalysts with tunable hydrogen oxidation kinetics in alkaline electrolyte. *J. Phys. Chem. C* **119**, 13481-13487 (2015).
8. St. John S, Atkinson RW, Unocic KA, Unocic RR, Zawodzinski TA, Papandrew AB. Platinum and palladium overlayers dramatically enhance the activity of ruthenium nanotubes for alkaline hydrogen oxidation. *ACS Catal.* **5**, 7015-7023 (2015).
9. Scofield ME, *et al.* Role of chemical composition in the enhanced catalytic activity of Pt-based alloyed ultrathin nanowires for the hydrogen oxidation reaction under alkaline conditions. *ACS Catal.* **6**, 3895-3908 (2016).
10. Jin Y, Chen F, Wang J, Guo L, Jin T, Liu H. Lamellar platinum–rhodium aerogels with superior electrocatalytic performance for both hydrogen oxidation and evolution reaction in alkaline environment. *J. Power Sources* **435**, 226798 (2019).
11. Hamo ER, *et al.* Carbide-supported PtRu catalysts for hydrogen oxidation reaction in alkaline electrolyte. *ACS Catal.* **11**, 932-947 (2021).
