# Supplementary material for: Atomic-precision Pt6 nanoclusters for enhanced hydrogen electro-oxidation
Source: Nat Commun. 2022 Mar 24;13:1596. doi: 10.1038/s41467-022-29276-7 (PMC8948276; doi:10.1038/s41467-022-29276-7)
Supplement: Supplementary file 3 — Description of Additional Supplementary Files [file 41467_2022_29276_MOESM3_ESM.pdf]

Description of Additional Supplementary Files

File name: Supplementary Movie 1

Description: The Supplementary Movie shows that once introducing the strong reducing agent $NaBH_4$ into the solution containing Pt precursor, abundant bubbles of H_2, which is the product of $NaBH_4$ decomposition and acts as active species in the subsequent reduction reaction, could be observed.
